# Normative cognition and the effects of a probiotic food intervention in first grade children in Côte d’Ivoire

**DOI:** 10.1038/s41598-022-23797-3

**Published:** 2022-11-14

**Authors:** Bonnie E. Brett, Habib O. Y. Doumbia, Bruno K. Koko, Frédéric Kouadio Koffi, Savorgnan E. Assa, Kollet Y. A. S. Zahé, Remco Kort, Wilbert Sybesma, Gregor Reid, Carolina de Weerth

**Affiliations:** 1 Department of Cognitive Neuroscience, Donders Institute for Brain, Cognition, and Behaviour, Radboud University Medical Center, Nijmegen, The Netherlands; 2 UFR Biosciences, Université Félix Houghouët-Boigny, Abidjan, Côte d’Ivoire; 3 Yoba For Life Foundation, Amsterdam, The Netherlands; 4 Department of Molecular Cell Biology, Vrije Universiteit Amsterdam, Amsterdam, The Netherlands; 5 Lawson Health Research Institute and Western University, London, Canada

**Keywords:** Human behaviour, Nutrition

## Abstract

The cognitive skills critical for success have largely been studied in Western populations, despite the fact that children in low- and middle-income countries are at risk to not reach their full developmental potential. Moreover, scientists should leverage recent discovery to explore means of boosting cognition in at-risk populations. This semi-randomized controlled trial examined normative cognitive development and whether it could be enhanced by consumption of a probiotic food in a sample of 251 4- to 7-year-old children in urban schools in Côte d’Ivoire. Participants completed executive functioning measures at baseline (T1) and 5 months later (T2). After T1, children in one school received a probiotic (*N* = 74) or placebo (*N* = 79) fermented dairy food every day they were in school for one semester; children in the other school (*N* = 98) continued their diet as usual. Children improved on all tests across time (Cohen’s d = 0.08–0.30). The effects of probiotic ingestion were inconclusive and are interpreted with caution due to socio-political factors affecting daily administration. Given the general feasibility of the study, we hope that it will serve as an inspiration for future research into child development and sustainable (health-promoting) interventions for school children in developing nations.

## Introduction

Executive functioning (EF) is an umbrella term for a set of skills including working memory and response inhibition^1^. EF and numeracy skills at school entry are critical components of academic success and contribute to other beneficial life outcomes. For example, deficits in EF in first graders (4–6 years old) are linked with later writing and mental health difficulties, whereas EF competence is linked with school achievement and social competence^2–4^. Numerical skills at the same age contribute to functional numeracy, or mathematical competence, 6 years later^5^. In turn, numeracy at later ages is negatively associated with self-reports of poor health and positively associated with higher personal wealth^5–7^. Despite recognition of the importance of these skills, their development is largely studied in white, educated, industrialized, rich, and democratic (WEIRD^8^) populations, limiting the generalizability of findings to a small subset of the world’s population. The current study examined normative cognitive development, and whether it is enhanced through probiotic supplementation in the form of a locally produced probiotic fermented milk product, in an urban sample of first grade children in Côte d’Ivoire.

### Cognition in the developing world

To date, studies examining the development of EF and other cognitive skills in low- and middle-income or LMIC (i.e., non-WEIRD) populations have been limited in both number and scope. Extant studies have tended to focus on very young children prior to school entry, specific populations (e.g., HIV-infected or malnourished children), parenting or academic interventions, and test development (e.g., Refs.^9–11^). Studies that do examine normative school-age cognition in LMICs find it linked with important life outcomes, underscoring the need to study cognition in diverse populations. For example, one study found positive associations between early executive functioning and academic achievement in 1480 4- to 7-year-old Kenyan children; another with 150 South African primary school children showed a correlation between early academic achievement and cognitive test scores and later school retention and survival in a longitudinal investigation^12,13^.

Despite the paucity of literature, there is growing interest in examining cognitive development in diverse populations, as it is widely recognized that children in LMICs, and particularly those in sub-Saharan Africa, are at risk to not reach their full development potential^14^. Children in LIMCs often face combined risk factors for suboptimal development, including parasitic infections, malnutrition, exposure to toxins, and food insecurity—all of which are linked with cognitive deficits (e.g., Refs.^10,15–18^). Understanding normative cognitive development in LMICs will help identify requirements to counter inequality in underserved communities and provide critical data for governments aiming to intervene when children are failing to reach their potential.

In addition to studying normative cognitive development, scientists should look to recent research and scientific discoveries to develop sustainable, low-cost, and accessible means to improve cognitive outcomes for at-risk populations. The literature points to at least two promising avenues to achieve this aim in developing contexts—nutrition supplementation and the use of probiotics.

### Nutrition supplementation

A recent systematic review examining school-based nutrition programs in sub-Saharan Africa found that affordable and practical programs demonstrate positive effects on cognitive performance in a number of domains^19^. Milk products in particular hold promise: a randomized controlled trial with first grade Kenyan children showed that children who received milk powder-supplemented stew each school day over two years had better end-term test scores than children who received oil-supplemented stew or no stew^20^. Additionally, a study in Ghana showed that adding milk powder to a school meal for nine months increased children’s executive functioning scores compared to a control group whose school meal was not supplemented^21^.

### Probiotics

Probiotics, defined as “live microorganisms that, when administered in adequate amounts, confer a health benefit on the host”^22^, may be another promising route to boost cognitive outcomes. Although no research has yet examined probiotic effects on cognition in LMICS, studies have shown health benefits such as reduced absorption of environmental toxins, reduced respiratory infections, and shortened duration of acute diarrhea, a common ailment in the developing world^16,23–25^. The latter two studies utilized *Lacticaseibacillus rhamnosus* GG, the strain from which a generic variant, *Lacticaseibacillus rhamnosus* yoba 2012, was developed by Yoba for Life Foundation^26^, which was used in the present study. It is possible that such documented health benefits would be indirectly linked with cognitive improvements in children by, for example, decreasing school absence.

Another route through which probiotics may induce cognitive improvements is through modulation of the microbiota-gut-brain axis. A recent review indicated that probiotic ingestion was associated with positive gut microbiota changes (e.g., more healthy and fewer unhealthy bacteria) in healthy older adults^27^, although this has not been replicated in children. However, in very young children, gut microbiota composition is associated with both cognition^28^ and brain functional connectivity^29^, indicating that positive alterations to the gut microbiota (i.e., through probiotic supplementation) may also influence cognitive function.

Empirically, the evidence for probiotic effects on cognition is small and somewhat inconsistent. While the animal literature points to positive probiotic effects on learning and memory (e.g., Ref.^30^), human studies, which generally involve probiotic supplementation for between 3 and 12 weeks, are more equivocal: one recent meta-analysis found evidence suggesting positive probiotic effects on cognition in humans^31^, whereas another did not^32^. A recent review reported that 21 out of the 25 studies in adult humans examined found a positive effect of probiotic consumption on cognition. In the same review, however, studies examining very young children tended to find no effects. Importantly, all occurred in children under 2 years of age, a time in which the brain is undergoing rapid development and potentially susceptible to a wide range of external influences^33^. Similarly, probiotic strains such as *Lacticaseibacillus rhamnosus* HN001 and *Bifidobacterium animalis* subsp. *lactis* HN019 were unassociated with cognitive improvements in infants^34^. However, a randomized placebo-controlled study of infants showed that *L. rhamnosus* GG (a strain similar to the one used in the current investigation) could have a benefit against attention deficit hyperactivity disorder and Asperger syndrome^35^. No studies have yet examined the effects of probiotic ingestion on cognition in school-aged children, which is surprising given that the safety record of this class of products is excellent for children of all ages (e.g., Ref.^36^). Based on the evidence presented above on outcomes and potential mechanisms, we hypothesize that *L. rhamnosus* yoba 2012, administered with a nutrient-rich food, may improve cognitive functioning in children from an economically stressed setting.

### The current study

This manuscript describes the results of a semi-randomized controlled trial with first grade children in Côte d’Ivoire. The first aim was to examine children’s normative cognitive gains, using free and widely available age-appropriate tests, over one semester at urban, economically-disadvantaged schools. We hypothesized that children would improve on all tests over one semester. The second aim was to determine whether regular intake of a traditional fermented milk-based probiotic product with millet, locally known as dêguê, that was enriched with the probiotic *L. rhamnosus* yoba 2012 could enhance cognitive scores compared to a non-enriched variant (placebo). We hypothesized that children who consumed the fermented probiotic product for one semester (approximately 16 weeks) would have higher scores at the end of the semester than children who consumed a placebo and children who followed their diet as usual, taking into account baseline scores. Finally, we exploratorily examined whether probiotic dose or regularity of consumption were related to test scores, but advanced no hypotheses about these.

## Methods

### Study design and participants

This semi-randomized controlled trial had three arms: probiotic, placebo, and diet as usual (DAU). We employed a semi-randomized design due to the ethical concern of giving some children but not others a food supplement in a single school. Accordingly, children in one school (the experimental school) comprised the probiotic and placebo arms randomized within classes, whereas children in the other school (the control school) comprised the DAU arm. A power analysis conducted with G*Power^37^ determined that 3 groups with at least 89 participants per group would give us 90% power to detect small to medium group mean differences. To plan for participant attrition and to increase power, we aimed to include at least 100 children in each group.

Children were recruited (via flyers sent home in backpacks, word of mouth, and two social events held at the schools) from first grade classes (level CP1 in the French/Ivorian education systems) in two closely-situated public primary schools in a low-income district of Abidjan, Côte d’Ivoire. All first-grade children were West-African and eligible for inclusion. In the experimental school, all available (i.e., currently enrolled in first grade) children were recruited (*N*_probiotic_ = 87, *N*_placebo_ = 82, *N*_DAU_ = 1). Given that schools were assigned by the government, we were unable to recruit additional children from nearby schools or switch to a school with more available children. In the control school, the first 100 children whose caregivers consented were recruited (*N*_DAU_ = 100), resulting in a total sample of 270 children.

Caregivers provided written informed consent for themselves and their children to take part in the study; children provided verbal assent prior to each round of testing. Caregivers who were able to read could take the consent form to read at their leisure and return at any time to sign up. Caregivers who were illiterate (the majority) had the consent form verbally explained page-by-page by a member of the research team. All caregivers were given the opportunity to pose questions before signing (or providing a thumbprint if unable to sign).

The study was approved by the “Comité National d'Ethique des Sciences de la Vie et de la Santé” (National Committee of Ethics of Life Sciences and Health) in Côte d’Ivoire and the ethics committee of the Social Science faculty of Radboud University in The Netherlands (project number: ECSW-2018-085R1). All methods were performed in accordance with the guidelines and regulations set forth by both committees, and approved procedures were not deviated from. The study was registered: AsPredicted #32143 (10/15/2019). To this registration we added a general question about changes in cognition over time. Additionally, not all variables mentioned in the registration were able to be used and analytic techniques were adapted due to missing data. In addition, we registered the trial with the Pan African Clinical Trials Registry: PACTR202207495830367. This registration was completed on June 18, 2022 and accurately reflects our work.

### Procedure

Caregivers (*N* = 231) completed a 20 to 30-min interview on family demographics and child characteristics either immediately after consenting or 1–4 months later via telephone. All efforts were made to ensure that the respondent was the primary caregiver of the child, but in some instances, this was not possible, and it was completed by someone else close to the child.

At baseline (T1) prior to randomization and at outcome (T2; 3–5 months later; mean = 4.89 months; standard deviation = 19.39 days), children completed cognitive measures and provided biological measures (height and weight, saliva and fecal samples) that are not part of the current study.

After most T1 testing was complete, experimental school children were randomized into probiotic and placebo groups stratified by class. Children were assigned numbers; a random number generator determined groups. Children enrolled after randomization (*N* = 11) were assigned on an alternating basis. For one semester, randomized children were given a probiotic or a placebo product each day they were in class. No child consumed the product prior to completing T1 testing. Children in the control school received no dietary intervention. Medical care was available at no cost to all participants during the study period.

### Dietary intervention

Probiotic and placebo dêguê (a well-known West African fermented milk product containing sugar and millet) were produced for this study by a local dêguê-producing company using standard hygiene practices including gloves and hair and shoe coverings. The company adapted their recipe to create a healthier product with 40% less sugar than their usual commercial product. Probiotic dêguê was fermented using *L. rhamnosus* yoba 2012 and *Streptococcus thermophilus* C106; placebo dêguê was fermented using only *S. thermophilus* C106. We chose *L. rhamnosus* yoba 2012 as it is a well-documented probiotic strain that has been shown to confer health benefits (e.g., Refs.^24,26,38^), whereas *S. thermophilus* alone, common in yogurt production worldwide, has not been examined specifically for its probiotic properties in humans (although it is often paired with other probiotics, due to its synergistic effects^26^). Ferments were provided to the company in labelled sachets provided by the Yoba-for-Life Foundation. Tests performed in the Netherlands confirmed that the sachets contained the expected bacteria. The local dêguê producer was trained on how to use the Yoba starter culture for the production of probiotic fermented dêguê. Final cell counts are expected to vary between 2-9E7 cfu/mL for *L. rhamnosus* yoba 2012 and 0.8-2E9 cfu/mL for *S. thermophilus* C106 (Ref.^26^, and see Ref.^39^ for the bacterial profile of *L. rhamnosus* yoba 2012 in a similar fermented dish). In-country tests of the product confirmed that it was safe for consumption. Adult consumers reported that the products were similar, but that the probiotic dêguê was slightly more sour.

Dêguê was served in locally purchased 125 mL cups with snap-on lids. Probiotic and placebo cups were indistinguishable other than a small label with a nearly-identical series of numbers on the side of the cup. Dêguê was delivered to schools in coolers on ice and distributed in classrooms. Children were monitored to ensure they only consumed their own dêguê.

### Measures

#### Child age and gender

Caregivers reported their child’s gender. Schools and caregivers provided birthdays. When reporters disagreed or provided incomplete data (e.g., a month and year but no day), a series of decisions was used to estimate ages (see Supplementary Material Section S1). Ages are presented as age in days at December 1, 2018.

#### Socioeconomic status (SES)

Caregivers endorsed which of 20 household items they owned, a common procedure for estimating SES in low-income contexts (e.g., Ref.^40^). The list of items was created in consultation with local researchers. Scores are the number of items endorsed; higher scores reflect higher SES.

#### Milliliters of product consumed

Each day, researchers recorded when children were present and consumed 125 mL of dêguê (which weighed 125 g). If a child did not finish a serving, their cup was weighed; the amount remaining was subtracted from 125 g to approximate how much they had eaten. Scores reflect the approximate total milliliters of dêguê consumed prior to T2 testing.

#### Regularity of product consumption

Children were defined as regular consumers when they consumed some dêguê on at least 2 days in nearly every week it was offered prior to testing.

#### Pen and paper cognitive tests

All cognitive tests were administered by trained Ivorian researchers in a semi-private area at the child’s school. Tests were administered in the same manner to all participants; parallel forms were used for the second administration of each. The Numeracy task was added to the end of the battery in T2 after researchers became aware of it at a conference. Children used a large marker to complete pen and paper tasks.

##### Cancellation task

A cancellation task was used to measure selective attention and processing speed (e.g., Ref.^41^). Children had 60 s to cross out as many target items (e.g., t-shirts) they could find out of 35 among a field of approximately 250 distractor items (e.g., other types of clothing). Scores are the number of correctly crossed items minus the number of incorrectly crossed items.

##### Numeracy task

To assess numerical magnitude understanding, children completed the Numeracy Screener^42^ at T2. The screener consists of two sections, a non-symbolic (dots) and a symbolic (numbers) section, completed in an identical manner. In the task, children saw pairs of dot clusters or numbers and were instructed to mark the larger of the two. Children completed a practice round with feedback and then had 60 s to complete as many items as they could out of 56. Children have two scores reflecting the number of correctly completed items for the dot and number sections, respectively.

#### Computerized cognitive tests

All computer tasks were adapted from the NIH EXAMINER battery^43^. Adaptations were completed in consultation with local partners after pilot testing and included French translation, simplification of language, removal of written instructions, addition of hand gestures, and increasing the size of the arrow keys using laminated arrows. The tasks were unchanged.

Children could only complete a computer task if they demonstrated proficiency in a practice trial. Children had three opportunities to pass the practice trial for each task before it was skipped. Further details about the measures and score generation can be found in Ref.^43^.

##### Flanker task

In this measure of response inhibition, children saw a horizontal line of five fish and were instructed to press the arrow key corresponding to the direction the middle fish was facing, which could be congruent or incongruent with the other fish (e.g., Ref.^44^). Children had to correctly respond to 6 out of 8 practice items to pass to the task.

The task consisted of 48 trials: 24 congruent and 24 incongruent. Automatically generated scores are on a 0–10 scale and reflect the speed and accuracy of children’s responses in incongruent trials. Higher scores indicate better performance.

##### Set shifting

In this task measuring cognitive flexibility, children were asked to match a colored shape (e.g., red rectangle) in the middle of the screen to one of two colored shapes (e.g., a blue rectangle and a red triangle) on the bottom of the screen using either a shape rule or a color rule (e.g., Ref.^45^). Children had to correctly respond to 12 out of 16 practice items to pass to the task.

The task consisted of 104 trials. In the first and second blocks of 20 trials each, children applied a single rule (e.g., shape or color) that changed from block one to block two. In the final 64 trials, the rule shifted from trial to trial. Automatically generated scores are on a 0–10 scale and reflect the speed and accuracy of children’s responses in shifting trials. Higher scores indicate better performance.

##### Go/no go

In the Continuous Performance Task, a Go/no go (GNG) task that measures sustained and selective attention and response inhibition, children pressed a button when they saw a target image displayed in a series of distractor images (e.g., Ref.^46^). Children had to correctly respond to 16 out of 20 practice items to pass to the task.

The task consisted of 104 trials. Automatically generated scores reflect the total number of times the child correctly pressed or did not press the button; higher scores indicate better performance.

### Analysis plan

#### Main and exploratory analyses

To examine whether children improved across time on cognitive tests (except Numeracy), we ran paired samples t-tests comparing T1 and T2 scores. To examine whether consumption of a fermented probiotic food product influenced cognition, we performed ANCOVA analyses predicting T2 cognitive performance using group as a predictor and T1 scores as a covariate. In addition, due to a disruption in probiotic administration (see “Results”), we also did exploratory dose–response and regularity analyses to examine whether higher doses or regular administration (as intended) were associated with cognitive outcomes. In the probiotic and placebo groups only, we used multiple regression to see if the amount of dêguê consumed or regularity of consumption influenced T2 scores, controlling for T1 scores and including the group × mL consumed/regularity interaction. Missingness was handled using complete case analysis, as is recommended for trials with large amounts of missing data^47^.

### Sensitivity analyses

We performed two types of sensitivity analysis. First, we a priori decided to include child gender, age, and SES as covariates and reran all analyses accordingly using complete case analysis. Second, for all tasks but the Numeracy task (whose missing data was negligible) we ran sensitivity analyses appropriate to their missing data. For the Cancellation task, we used multiple imputation to replace missing task and covariate values and reran analyses with and without covariates. For all computer tasks, given that data were missing not at random, we performed worst-case scenario analyses with and without covariates in which we calculated mean scores by group and replaced missing values with a value two standard deviations below the mean^47^.

## Results

### Preliminary analyses

**Figure 1:**
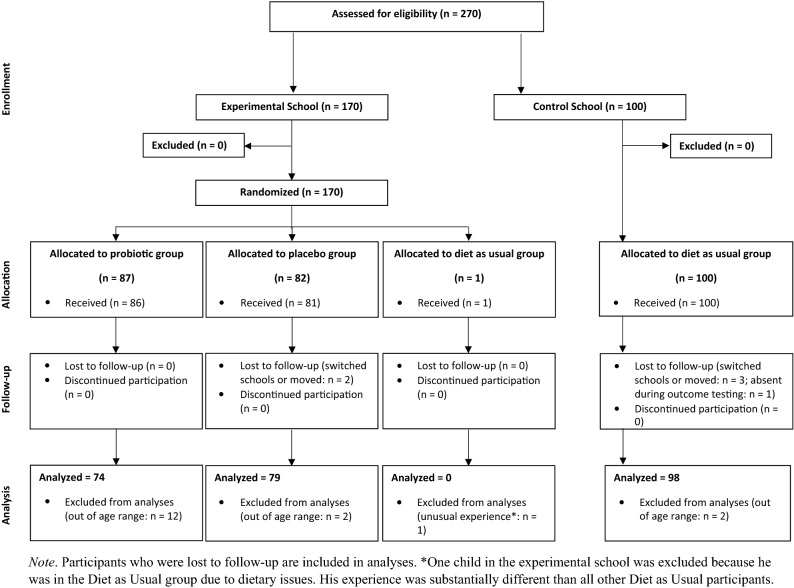
Consort participant flow diagram. Participants who were lost to follow-up are included in analyses. *One child in the experimental school was excluded because he was in the Diet as Usual group due to dietary issues. His experience was substantially different than all other Diet as Usual participants.

Between December 2018 and February 2019, 270 children enrolled in the study (*N*_probiotic_ = 87, *N*_placebo_ = 82, *N*_DAU_ = 101) from two schools in the Koumassi district of Abidjan. Current analyses exclude 16 children outside the target age range, two who enrolled but did not participate, and one in the experimental school who was placed in the DAU group due to medical issues, leaving a final sample of 251 4- to 7-year-old children (*N*_probiotic_ = 74, *N*_placebo_ = 79, *N*_DAU_ = 98). Six children left the study prior to outcome data collection (*N*_placebo_ = 2, *N*_DAU_ = 4) but are included in analyses (Fig. 1). Demographic differences between the experimental and control schools revealed that control school caregivers reported higher SES than experimental school caregivers (Table 1).

**Table 1:** Sample demographics and school comparison.

	Sample (n = 251)	By group			
		Experimental school		Control school	
		Probiotic (n = 74)	Placebo (n = 79)	Diet as usual (n = 98)	
Variable (range)	*M (SD)* *N*	*M (SD)* *N*	*M (SD)* *N*	*M (SD)* *N*	Test statistic^a^
Gender (% girls)	55.38%	60.81	46.84	58.16	0.505^b^
Age in years (4.09–6.98)	5.51 (0.61) 218	5.59 (0.58) 59	5.46 (0.67) 68	5.49 (0.59) 89	0.372^c^
SES (3–17)	9.44 (2.52) 234	8.56 (2.35) 66	9.01 (2.51) 74	10.40 (2.33) 94	− 5.03*^c^
mL dêguê consumed (970–6344)	4172.02 (1083.98) 151	4169.69 (968.67) 74	4174.26 (1190.23) 77		
Consistent consumers (% consistent)	28.10%	22.97%	32.91%		

*M* Mean, *SD* Standard deviation. ^a^Test statistics are for differences between the experimental school and control school. ^b^χ^2^ tests for categorical data. ^c^Independent samples t-tests for normally distributed data. *SES* Socioeconomic status, *mL* Milliliters.

*p < 0.001.

Probiotic and placebo dêguê were provided to experimental school participants every Monday through Friday from January 14, 2019 to May 15, 2019 except 20 days due to production issues, national holidays, and school exams. In the remaining 68 days, children could receive between 53 and 68 portions prior to T2 testing, depending on when they were tested (provision of dêguê continued until the end of testing). Thirty-three of these possible days coincided with a nationwide teacher strike in which schools were closed but the research team was present and attempted contact to encourage caregivers to bring children to school for the consumption of dêguê. The mean number of days children consumed dêguê was 33.65 (SD = 10.97) and equal between groups (*M*_probiotic_ = 33.64, *SD* = 10.31; *M*_placebo_ = 33.66, *SD* = 11.69). Experimental groups consumed similar quantities of dêguê over the study period (*t* = − 0.03; *p* = 0.979) and had a statistically equivalent number of “regular” consumers (χ^2^ = 1.87, *p* = 0.172; Table 1). More than 95% of cups given were finished and no adverse health effects of the product were reported.

Missing data were rare for paper tasks but common for computer tasks as many children did not pass the practice trials (Table 2). For this reason, computer task analyses are considered exploratory and are not representative of the sample as a whole.

**Table 2:** Percentages of missing data by task.

	T1% missing				T2% missing^a^				
	Sample	Probiotic	Placebo	DAU		Sample	Probiotic	Placebo	DAU
**Paper tasks**									
Cancellation^b^	0	0	0	0		6.78	12.16	5.06	4.08
Numeracy	n.a	n.a	n.a	n.a		2.39	0	2.53	4.08
**Computer tasks**^**c**^									
Flanker	67.33	63.51	73.42	64.29		54.98	55.41	63.29	47.96
Set shifting	73.31	71.62	81.01	68.37		60.16	56.76	70.89	54.08
Go/no-go	56.57	60.81	68.35	43.88		44.22	40.54	58.23	35.71

*DAU* Diet as usual, *n.a.* not administered.

^a^6 lost to follow-up; ^b^11 lost due to procedure change; ^c^primary reason for missing response is failure to pass practice rounds of test (< 0.5% of responses lost due to computer error).

### Main analyses

Analyses were conducted in SPSS Version 25. Sensitivity analyses are mentioned when they differed from main or exploratory analyses.

#### Improvement across one semester

**Figure 2:**
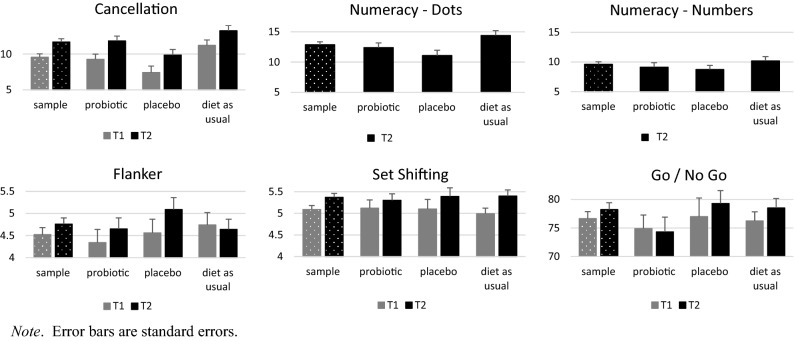
Mean cognitive test scores at T1 and T2 by group. Error bars are standard errors.

Children improved in all tasks across time (Cohen’s d = 0.08–0.30; Fig. 2; Table 3; Supplemental Materials, Table S1).

**Table 3:** Descriptive statistics by group, tests of improvement over time, and group effects.

	T1	T2	Test statistic	Estimated mean difference (95% CI)
	*N*; *M *(*SD*)	*N*; *M *(*SD*)	*t*/*F* (df; *p*)	
**Cancellation**				
Range	− 18 to 30	− 18 to 29		
Sample	251; 9.44 (7.44)	234; 11.76 (6.78)	− **4.38**^**a**^** (233; < 0.001)**	− 2.26 [− 3.28, − 1.24]
Probiotic	74; 9.26 (6.18)	65; 11.82 (5.45)	2.55^b^ (2, 230; 0.081)	
Placebo	79; 7.42 (8.04)	75; 9.85 (6.74)		
DAU	98; 11.21 (7.45)	94; 13.24 (7.31)		
**Numeracy—dots**				
Range		0–33		
Sample		245; 12.75 (7.55)		
Probiotic		74; 12.39 (6.73)	**4.34**^**b**^** (2, 242; 0.014)**	
Placebo		77; 11.08 (7.75)		
DAU		94; 14.40 (7.73)		
**Numeracy—numbers**				
Range		0–32		
Sample		245; 9.40 (6.58)		
Probiotic		74; 9.12 (6.56)	1.13^b^ (2, 242; 0.324)	
Placebo		77; 8.73 (6.02)		
DAU		94; 10.18 (7.01)		
**Flanker**				
Range	1.05–7.71	1.67–7.86		
Sample	82; 4.57 (1.55)	113; 4.76 (1.54)	**− 2.33**^**a**^** (51; 0.024)**	− 0.54 [− 1.01, − 0.07]
Probiotic	26; 4.34 (1.53)	33; 4.65 (1.43)	0.20^b^ (2, 48; 0.818)	
Placebo	21; 4.56 (1.43)	29; 5.09 (1.46)		
DAU	35; 4.74 (1.65)	51; 4.64 (1.66)		
**Set shifting**				
Range	3.03–7.21	3.38–7.75		
Sample	67; 5.06 (0.80)	100; 5.37 (0.91)	**− 2.18**^**a**^** (48; 0.034)**	− 0.33 [− 0.64, − 0.03]
Probiotic	21; 5.12 (0.89)	32; 5.30 (0.85)	0.23^b^ (2, 45; 0.796)	
Placebo	15; 5.10 (0.86)	23; 5.39 (0.94)		
DAU	31; 4.99 (0.74)	45; 5.40 (0.94)		
**Go/no go**				
Range	18–95	35–98		
Sample	109; 76.06 (13.16)	140; 77.39 (14.51)	**− 2.89**^**a**^** (90; 0.005)**	− 4.62 [− 7.79, − 1.45]
Probiotic	29; 74.90 (12.80)	44; 74.32 (17.18)	0.21^b^ (2, 87; 0.811)	
Placebo	25; 77.00 (16.37)	33; 79.30 (12.91)		
DAU	55; 76.24 (11.87)	63; 78.52 (13.09)		

^a^Paired samples t-test comparing scores at T2 with those of T1 for the group as a whole; ^b^ANCOVA examining group difference in T2 scores with T1 scores included as covariate (except Numeracy).

#### Probiotic effects

##### Cancellation

There were no group effects on T2 Cancellation scores (Table 3; Fig. 2). Sensitivity analyses revealed that the placebo group mean was significantly lower than the grand mean; this effect disappeared with the inclusion of imputed covariates (Supplemental Materials, Table S2).

##### Numeracy

An ANOVA uncovered group effects for dot scores (Table 3, Fig. 2). Fisher’s LSD post-hoc comparison revealed that the placebo group had significantly lower scores than the DAU group (p value). The group effect became nonsignificant with the inclusion of covariates (Supplemental Materials, Table S2). There was no group effect predicting number scores (Table 3; Fig. 2; Supplemental Materials, Table S2).

##### Computer tasks

There were no group effects predicting T2 Flanker or Set shifting scores in any analysis or for T2 GNG scores in complete case analyses with or without covariates. Worst-case scenario sensitivity analyses revealed that the probiotic group had lower scores than the placebo and DAU groups (Table 3; Fig. 2; Supplemental Materials, Table S3).

### Exploratory analyses

#### Dose response

##### Cancellation

Milliliters consumed was not significantly associated with T2 Cancellation scores accounting for T1 scores, but its main effect trended toward significance in all analyses (Table 4; Supplemental Materials, Table S4).

**Table 4:** Regression analyses predicting test scores from mL Dêguê consumed and regularity.

	mL of dêguê consumed				Regularity of consumption		
	*ß*	*b*	*p*		*ß*	*B*	*p*
**Cancellation**	***F* (4, 139) = 4.32, *p* = 0.003**				***F* (4, 139) = 5.21, *p* = 0.001**		
Constant		8.39	< 0.0001	Constant		9.26	< 0.0001
T1 cancel	0.24	0.30	0.003	T1 cancel	0.23	0.20	0.006
Group	− 0.14	− 1.70	0.082	Group	− 0.16	− 2.00	0.055
mL consumed	0.16	0.001	0.058	Regularity	0.20	2.92	0.021
Gp*mLc	0.01	− 0.001	0.878	Gp*R	0.07	2.00	0.430
*R*^2^	0.34			*R*^2^	0.37		
**Numeracy-dots**	*F* (3, 147) = 0.86, *p* = 0.462				*F* (3, 147) = 1.04, *p* = 0.375		
Constant		11.71	< 0.0001	Constant		11.71	< 0.0001
Group	− 0.09	− 1.32	0.269	Group	0.11	− 1.45	0.187
mL consumed	0.09	0.001	0.267	Regularity	0.02	1.78	0.225
Gp*mLc	0.02	0.000	0.847	Gp*R	− 0.10	0.73	0.788
*R*^2^	0.13			*R*^2^	0.02		
**Numeracy-#s**	*F* (3, 147) = 0.68, *p* = 0.564				*F* (3, 147) = 0.17, *p* = 0.915		
Constant		8.92	< 0.0001	Constant		8.90	< 0.0001
Group	− 0.03	− 0.39	0.701	Group	− 0.03	− 0.33	0.784
mL consumed	0.01	0.000	0.916	Regularity	− 0.05	− 0.65	0.578
Gp*mLc	0.11	0.001	0.185	Gp*R	0.02	0.67	0.776
*R*^2^	0.12			*R*^2^	0.004		
**Flanker**^a^	***F* (4, 22) = 3.21, *p* = 0.032**						
Constant		1.75	0.109				
T1 flanker	0.57	0.77	0.004				
Group	0.002	0.01	0.992				
mL consumed	0.08	0.00	0.642				
Gp*mLc	0.11	0.00	0.539				
*R*^2^	0.37						
**Set shifting**^a^	*F* (4, 20) = 0.25, *p* = 0.905						
Constant		4.68	0.001				
T1 set shifting	0.13	0.13	0.563				
Group	− 0.12	− 0.24	0.590				
mL consumed	0.16	0.000	0.496				
Gp*mLc	0.002	0.000	0.992				
*R*^2^	0.05						
**Go/no go**	*F* (4, 43) = 1.83, *p* = 0.140				*F* (4, 43) = 1.02, *p* = 0.410		
Constant		68.23	< 0.0001	Constant		64.94	< 0.0001
T1 go/no go	0.19	0.16	0.203	T1 go/no go	0.24	0.20	0.109
Group	0.07	1.69	0.643	Group	0.04	1.11	0.769
mL consumed	− 0.07	− 0.001	0.645	Regularity	− 0.05	− 1.51	0.720
Gp*mLc	0.27	0.01	0.075	Gp*R	0.11	6.17	0.468
*R*^2^	0.15			*R*^2^	0.09		

*ß* = standardized regression coefficient; *b* = regression coefficient; *R*^*2*^ = total variance explained by the model. #s = numbers. mL = milliliters. Gp*mLc = group by milliliters consumed interaction. Gp*R = group by regularity classification interaction. ^a^Too few regular consumers to examine regularity effects. Significant models are bolded.

##### Numeracy

Models predicting dot and number scores using milliliters consumed were not significant (Table 4). The model predicting number scores became significant with the inclusion of covariates, but no predictors other than age reached significance (Supplemental Materials, Table S4).

##### Flanker

Neither group nor milliliters consumed were significant in any models predicting T2 Flanker scores (Table 4; Supplemental Materials, Table S4).

##### Set shifting

No model predicting T2 Set shifting scores using milliliters consumed were significant except a worst-case scenario sensitivity analysis without covariates in which no predictors other than T1 scores reached significance (Table 4; Supplemental Materials, Table S4).

##### Go/no go

The model predicting T2 Go/no go scores was not significant (Table 4), but all sensitivity analysis models were (Supplemental Materials; Table S4). Complete case analysis with covariates revealed a significant interaction such that milliliters consumed was negatively associated with Go/no go scores in the probiotic group only. In the worst-case scenario sensitivity analyses, the interaction was not significant, but probiotic group scores were lower than those for the placebo group (Supplemental Materials; Table S4).

#### Regularity effects

##### Cancellation

There was a main effect of regularity on T2 scores accounting for T1 scores, with regular consumers having higher scores than non-regular consumers (Table 4). The effect trended toward significance in sensitivity analyses using multiple imputation (Supplemental Materials; Table S5).

##### Numeracy

Models predicting dot and number scores using regularity classification were not significant (Table 4). Models with covariates were significant, but neither group nor regularity classification effects were (Supplemental Materials; Table S5).

##### Flanker

There were fewer than 10 regular consumers with Flanker scores, so we ran this only as a worst-case scenario analysis and found no effects (Supplemental Materials; Table S5).

##### Set shifting

There were fewer than 10 regular consumers with set shifting scores, so we ran this only as a worst-case scenario analysis and found no effects (Supplemental Materials; Table S5).

##### Go/no go

The model predicating T2 Go/no go scores with regularity classification and group was not significant (Table 4). The models were significant in worst-case scenario sensitivity analyses and in both, group significantly predicted Go/no go scores with probiotic scores being lower than placebo (Supplemental Materials; Table S5).

## Discussion

This semi-randomized controlled trial examined cognitive change over time and the effects of a probiotic food intervention on first-graders’ cognitive outcomes. Children improved across one semester in all tasks, showing significantly higher scores and more children able to complete tasks at T2. Examinations of probiotic effects were discrepant between main and sensitivity analyses but largely indicated no effect of the probiotic on cognition in the present sample.

Notably, children showed improvement in all tasks despite a disrupted semester in which schools were closed for 5 weeks (and then closed every Wednesday after courses resumed) due to a nationwide teacher strike. That children improved is consistent with the notion that aspects of executive functioning improve across one semester (e.g., Ref.^48^) and suggests that such improvements are not entirely dependent on formal schooling. This study provides a first look into normative cognitive development over time in first grade children in Côte d’Ivoire. Future work examining how gains are amplified in an uninterrupted school year will add to the growing body of literature on cognitive development in LMICs. In addition, examining how this population compares with other populations in Abidjan and surrounding areas (e.g., higher SES; more rural) could inform the field on factors contributing to cognitive development in LIMCS.

In the current sample, there were no apparent effects of the probiotic on cognition. This may be in part because the consumption of dêguê was substantially interrupted due to the 5-week teacher strike during which less than 20% of participants came to school to consume dêguê. In addition, analyses must be considered critically as we were unable to reach our target sample of 300 participants, leading to power concerns, particularly in the computer tasks which also had large amounts of missing data. Interestingly, although results did not reach significance, our most consistent analyses point toward possible effects of milliliters consumed and regularity on cancellation scores (in the probiotic and placebo groups only). While these trends could also be explained by unexamined child characteristics, such as agreeableness (e.g., willingness to consume the product and put forth effort on tests), we note that the majority of children consumed all dêguê offered to them, so this seems unlikely. Moreover, we also found a potential (i.e., only apparent in sensitivity analyses) effect in the opposite direction, with children who consumed more (both more mL consumed and more consistently) probiotic having lower Go/no go scores than children in the placebo group. While we considered all computer tasks to be both exploratory and non-representative of the sample as a whole (due to the large amount of missing data), this puzzling finding was consistent across both analyses and deserves consideration in future work. Most important, consistent and prolonged administration will be necessary to draw strong conclusions about probiotic effects on cognitive outcomes. Indeed, such studies are warranted given recent evidence pointing to probiotic effects on attention tasks in Thai children (6–12-year-olds) after prolonged (12 weeks) administration^49^. Moreover, multiple reviews published since this study was conducted have found evidence for a potential role of probiotics and prebiotics in improved cognitive function in humans^50–52^. Importantly, we note that all studies finding effects engaged in uninterrupted and/or prolonged (12 or more weeks) probiotic administration, underscoring the importance consistent administration will play in future investigations.

This ambitious study was the first of its type in Côte d’Ivoire and provided valuable insight, as did a previous study in Uganda with the same probiotic strains and another in Tanzania using a different probiotic strain^38,53^. In the current study, children appreciated the dêguê (i.e., 95% consumed the whole portion every time), and the product was easily produced and affordable, costing only 0.39 USD per 125 mL. Approaches using a similar fermented dairy product including the same strains tested here have shown that regular consumption can decrease intestinal infections, upper respiratory tract infections, and angina, and that their administration is feasible on a large scale^24,38^. Understanding the full range of benefits such probiotics may provide is a worthy goal, given their safety, availability, ease of use, and acceptance in diverse populations. In particular, due to a long tradition of consuming fermented foods in African culture, some scholars suggest that the local production and distribution of affordable probiotics holds promise to both empower women through small enterprises and positively affect the health of probiotic consumers^54^. Given the equivocal nature of our results (due to probiotic administration disruption and measurement difficulties) researchers should continue to examine whether affordable, locally produced, health-promoting foods can enhance also cognitive development, particularly in at-risk populations.

Despite challenges, this study has strengths. It was a three-armed semi-randomized controlled trial with very low attrition and high compliance. Children participated readily in the cognitive testing sessions and enjoyed the locally produced dêguê. Given large amounts of missing data, two types of sensitivity analyses leant support to the findings. Limitations of the present study are acknowledged as follows: first, as mentioned, it was not possible to deliver the probiotic food intervention 5 days a week during 4 consecutive months as planned. Second, although the study was designed for first grade (CP1) and hence piloted in 6- to 7-year-old children, the mean participant age was 5.5 years, which was unanticipated. This precluded some aspects of data collection (e.g., a test of working memory that required counting; self-reports of health) intended for use in the current investigation.

The breadth of ages in the current sample also brings up some important considerations for future work examining cognition in similar populations. LMIC researchers in schools might prepare for a substantially larger age range than would be expected per grade level and pilot in a wide range of ages to ensure test feasibility beyond what previous research has shown. The EXAMINER manual reports that 96.5% of 3–7-year-olds were able to complete the tasks used in the current study and indeed, much literature indicates that tasks such as the ones used in the present investigation can be used with younger populations. However, given that our majority five-year-old population struggled with the computer (but not the paper) tasks, researchers might also consider using non-digital versions in future work examining similar populations (e.g., Ref.^55^). Notably, the EXAMINER was normed in populations accustomed to electronic devices^43^. Evidence using touch screen tablets shows that electronic assessments are feasible for use in school aged children in LMICs, but we found that computerized assessments, where children have to link concepts on a screen to one or two keys on a keyboard, were too complicated for many participants, despite instructional adaptations designed to make them clearer (e.g., Ref.^56^). This study adds to the work of Willoughby et al.^9^ addressing the need to develop low cost and accessible ways of testing early cognition in LMICs by noting that computerized assessments (as opposed to tablet based) may prove too complicated for use in certain contexts. In addition, researchers may consider interviewing teachers to ascertain the academic ability of their participants before implementing measures that rely on letter and number recognition.

This study is a promising step in a research agenda aimed at examining cognitive development in LMIC contexts. We add to the literature by demonstrating that economically disadvantaged children improved in multiple domains of executive functioning across one semester, and by discussing some necessary considerations of using computerized cognitive assessments in a single grade level in French-speaking West Africa. These considerations will be valuable for researchers intending to conduct similar work in like settings. We also note that, despite significant study disruption due to socio-political factors, the study was well received by both the participants and the community. Given the general feasibility of the study, we hope that it may serve as an inspiration for future research into child development and into sustainable (health-promoting) interventions for school children in developing nations.

## Supplementary Information

Supplementary Information 1.

Supplementary Information 2.

## Data Availability

Data Sharing: We did not have participant consent to store deidentified data in an online repository. Deidentified participant data underlying the reported results will be available upon publication (no end date) to researchers with a methodologically sound proposal for use. Proposals should be directed to carolina.deweerth@radboudumc.nl.
