# Supplementary material for: Normative cognition and the effects of a probiotic food intervention in first grade children in Côte d’Ivoire
Source: Sci Rep. 2022 Nov 14;12:19491. doi: 10.1038/s41598-022-23797-3 (PMC9663712; doi:10.1038/s41598-022-23797-3)
Supplement: Supplementary file 1 — Supplementary Information 1. [file 41598_2022_23797_MOESM1_ESM.docx]

**S1. Method**

**Calculating the Age Variable**

Schools and caregivers provided birthdays. When reporters disagreed, information provided by the school was used. When reporters only provided a year, the child’s age was calculated as if their birthday were July 1 of the year indicated (*N* = 34). When reports provided only a month and year, the child’s age was calculated as if their birthday were the first day of the month and year provided (*N* = 3). If reporters only provided a month and day or only a month, we considered the birthday to be missing. We discarded one birthday that seemed implausible (i.e., parents reported an age of 15 years, but the child was clearly much younger).

When children had no birthday (or the parents reported something out of our age range that did not agree with the school report), the primary investigator watched videos and flagged children who looked younger than 4 or older than 7. Then we cross referenced this list with a list of sample height and weight outliers. When children were flagged and they were an outlier, we discarded them (*N* = 3). Since there were no outliers on the lower end of the scale, we also cross referenced the list with the five lowest height and weight values and discarded one child who was likely younger than 4.
