# Supplementary material for: Normative cognition and the effects of a probiotic food intervention in first grade children in Côte d’Ivoire
Source: Sci Rep. 2022 Nov 14;12:19491. doi: 10.1038/s41598-022-23797-3 (PMC9663712; doi:10.1038/s41598-022-23797-3)
Supplement: Supplementary file 2 — Supplementary Information 2. [file 41598_2022_23797_MOESM2_ESM.docx]

**S2. Results**

*Table S1*. Sensitivity Analyses of Improvement Across Time

|  | *t* (*p*) | Estimated mean difference [95% CI] |
| --- | --- | --- |
| Cancellation | **-4.53 (.000)** ^a^ | -2.28 [-3.26, -1.29] |
| Flanker | **-4.61 (.000)** ^b^ | -.41 [-.55, -.27] |
| Set shifting | **-5.59 (.000)** ^b^ | -.62 [-.88, -.36] |
| Go / no go | **-4.04 (.000)** ^b^ | -4.65 [-6.73, -2.57] |

*Note*. ^a^ pooled result of multiple imputation analysis. ^b^ result of worst-case-scenario analysis.

*Table S2.* Sensitivity Analyses of Group Effects on Paper Task Scores

|  | + covariates ^a^ | Imputation ^b^ | | Imputation + covariates ^b^ | |
| --- | --- | --- | --- | --- | --- |
|  | *F* (df; *p*) |  | *b* (*p*) |  | *b* (*p*) |
| Cancellation | .635 (2, 182; .531) | Constant | **8.52 (.000)** | Constant | -8.47 |
|  |  | T1 Cancel | .**33 (.000)** | T1 Cancel | **.27 (.000)** |
|  |  |  |  | Age | **.01 (.000)** |
|  |  |  |  | Gender | .69 (.467) |
|  |  |  |  | SES | .18 (.348) |
|  |  | DAU | .90 (.110) | DAU | .58 (.360) |
|  |  | Placebo | **-1.18 (.045)** | Placebo | -.44 (.504) |
| Numeracy - dots | 1.65 (2, 191; .195) |  |  |  |  |
| Numeracy - numbers | 1.34 (2, 191; .264) |  |  |  |  |

*Note*. ^a^ ANCOVA examining group difference in T2 scores with age, gender, SES, and T1 scores (for Cancellation only) included as covariates. ^b^ Pooled results of multiple imputation analyses with effect coded groups.

*Table S3.* Sensitivity Analyses of Group Effects on Computer Task Scores

|  | + covariates | | Worst-Case-Scenario | | Worst-Case-Scenario + covariates | |
| --- | --- | --- | --- | --- | --- | --- |
|  | *N* | *F* (df; *p*) | *N* | *F* (df; *p*) | *N* | *F* (df; *p*) |
| Flanker | 44 | 2.13 (2, 37; .133) | 251 | .37 (2, 247); .690 | 201 | 1.95 (2, 194); .145 |
| Set shifting | 39 | .45 (2, 32; .670) | 251 | 1.23 (2, 247); .293 | 201 | .93 (2, 194); .395 |
| Go / no go | 71 | .04 (2, 64; .957) | 251 | **4.00 (2, 247); .031** | 201 | **4.23 (2, 194); .016** |

*Note*. All are ANOVA / ANCOVA examining group difference in T2 scores. Covariates are age, gender, SES, and T1 scores.

*Table S4*. Sensitivity Analyses Predicting Test Scores From mL Dêguê Consumed

|  | + covariates | | |  | Sensitivity | | |  | Sensitivity + covariates | | |
| --- | --- | --- | --- | --- | --- | --- | --- | --- | --- | --- | --- |
|  | *ß* | *b* | *p* |  | *ß* | *b* | *p* |  | *ß* ^c^ | *b* | *p* |
| Cancellation | ***F* (7, 98) = 6.93, *p* = .039** | | |  | ***F* (4, 146) = 4.19 – 6.51,**  ***p* = .000 – .003** ^a^ | | |  | **F (7, 106) = 2.34 – 3.50,**  ***p* = .002 – .029** ^a^ | | |
| Constant |  | -2.49 | **.000** |  |  | 8.80 | .000 |  |  | -3.79 | .514 |
| Age | .20 | .01 | **.000** |  |  |  |  |  | .18 – .21 | .01 | **.032** |
| Gender | .07 | .79 | .103 |  |  |  |  |  | .02 – .09 | .72 | .543 |
| SES | .04 | .11 | **.014** |  |  |  |  |  | .04 – .10 | .19 | .420 |
| T1 cancel | .19 | .16 | .552 |  | .24 – .30 | .24 | **.001** |  | .21 – .09 | .19 | **.011** |
| Group | -.03 | -.39 | .740 |  | -.16 – -.12 | -1.72 | .089 |  | -.08 – -.02 | -.59 | .610 |
| mL consumed | .19 | .001 | .068 |  | .11 – .20 | .001 | .101 |  | .14 – .21 | .001 | .059 |
| Gp*mLd | -.03 | .000 | .799 |  | -.04 – .07 | .000 | .809 |  | -.11 – .02 | -.001 | .575 |
| *R*^2^ |  |  |  |  |  |  |  |  |  |  |  |
| Numeracy- dots | *F* (6, 107) = 2.15, *p* = .053 | | | | | | |  |  |  |  |
| Constant |  | -11.08 | .115 |  |  |  |  |  |  |  |  |
| Age | .31 | .01 | .001 |  |  |  |  |  |  |  |  |
| Gender | -.08 | -1.14 | .412 |  |  |  |  |  |  |  |  |
| SES | .11 | .35 | .226 |  |  |  |  |  |  |  |  |
| Group | -.04 | -.55 | .692 |  |  |  |  |  |  |  |  |
| mL consumed | .07 | .000 | .494 |  |  |  |  |  |  |  |  |
| Gp*mLd | -.03 | .000 | .749 |  |  |  |  |  |  |  |  |
| *R*^2^ | .11 |  |  |  |  |  |  |  |  |  |  |
| Numeracy- #s | ***F* (6, 107) = 2.69, *p* = .018** | | | | | | |  |  |  |  |
| Constant |  | -10.38 | .075 |  |  |  |  |  |  |  |  |
| Age | .32 | .01 | .**001** |  |  |  |  |  |  |  |  |
| Gender | -.18 | -2.27 | .051 |  |  |  |  |  |  |  |  |
| SES | .11 | .28 | .245 |  |  |  |  |  |  |  |  |
| Group | .002 | .02 | .985 |  |  |  |  |  |  |  |  |
| mL consumed | -.04 | .000 | .647 |  |  |  |  |  |  |  |  |
| Gp*mLd | .07 | .001 | .471 |  |  |  |  |  |  |  |  |
| *R*^2^ | .13 |  |  |  |  |  |  |  |  |  |  |
| Flanker | ***F* (4, 18) = 3.25, *p* = .026** | | |  | ***F* (4, 146) = 3.04, *p* = .019** ^b^ | | |  | ***F* (6, 107) = 2.69, *p* = .018** ^b^ | | |
| Intercept |  | -3.59 | .343 |  |  | 2.58 | .**000** |  |  | -2.63 | .094 |
| Age | .12 | .001 | .512 |  |  |  |  |  | .28 | .002 | .**002** |
| Gender | .10 | .31 | .604 |  |  |  |  |  | -.06 | -.21 | .505 |
| SES | .33 | .27 | .089 |  |  |  |  |  | .15 | .11 | .099 |
| T1 flanker | .68 | .91 | .**002** |  | .23 | .25 | .**004** |  | .33 | .35 | .**000** |
| Group | -.01 | -.02 | .973 |  | .05 | .18 | .497 |  | .004 | .01 | .963 |
| mL consumed | .01 | .000 | .955 |  | .02 | .000 | .789 |  | -.01 | .000 | .957 |
| Gp*mLd | .08 | .000 | .666 |  | .14 | .000 | .092 |  | .12 | .000 | .192 |
| *R*^2^ | .60 |  |  |  | .08 |  |  |  | .21 |  |  |
| Set shifting | *F* (7, 10) = 1.69, *p* = .218 | | |  | ***F* (4, 146) = 3.59, *p* = .008** ^b^ | | |  | *F* (7, 106) = 1.10, *p* = .369 ^b^ | | |
| Intercept |  | .36 | .894 |  |  | 3.10 | .**000** |  |  | 3.26 | .003 |
| Age | .57 | .002 | .083 |  |  |  |  |  | -.02 | .000 | .803 |
| Gender | .37 | .62 | .134 |  |  |  |  |  | -.03 | -.05 | .794 |
| SES | .22 | .12 | .406 |  |  |  |  |  | .10 | .04 | .285 |
| T1 set shifting | -.26 | -.25 | .325 |  | .25 | .29 | .**002** |  | .17 | .21 | .070 |
| Group | -.05 | -.08 | .849 |  | -.11 | -.22 | .177 |  | -.13 | -.27 | .177 |
| mL consumed | -.22 | .000 | .486 |  | .06 | .000 | .481 |  | .04 | .000 | .678 |
| Gp*mLd | -.05 | .000 | .839 |  | .08 | .000 | .341 |  | .11 | .000 | .254 |
| *R*^2^ | .54 |  |  |  | .09 |  |  |  | .07 |  |  |
| Go / no go | ***F* (7, 27) = 2.69, *p* = .018** | | |  | ***F* (4, 146) = 15.34, *p* = .000** ^b^ | | |  | ***F* (7, 106) = 6.37, *p* = .000** ^b^ | | |
| Intercept |  | 42.23 | .191 |  |  | 28.10 | .**000** |  |  | 35.54 | .**026** |
| Age | .15 | .01 | .417 |  |  |  |  |  | -.05 | -.004 | .600 |
| Gender | -.05 | -1.31 | .785 |  |  |  |  |  | -.02 | -.81 | .793 |
| SES | .19 | 1.15 | .287 |  |  |  |  |  | .04 | .30 | .642 |
| T1 go / no go | .11 | .10 | .557 |  | .537 | .61 | **.000** |  | .52 | .57 | **.000** |
| Group | .04 | 1.02 | .830 |  | .192 | 7.16 | **.007** |  | .24 | 8.75 | **.006** |
| mL consumed | -.09 | -.001 | .645 |  | -.066 | -.001 | .355 |  | -.09 | -.001 | .301 |
| Gp*mLd | .40 | .01 | .**040** |  | -.012 | .000 | .868 |  | .00 | .000 | .999 |
| *R*^2^ | .26 |  |  |  | .30 |  |  |  | .30 |  |  |

*Note*. ^a^ Pooled results of multiple imputation analysis. ^b^ Worst-case-scenario analysis.

*Table S5*. Sensitivity Analyses Predicting Test Scores from Regularity

|  | + covariates | | | |  | Sensitivity | | |  | Sensitivity + covariates | | |
| --- | --- | --- | --- | --- | --- | --- | --- | --- | --- | --- | --- | --- |
|  | *ß* | | *b* | *p* |  | *ß* | *b* | *p* |  | *ß* ^c^ | *b* | *p* |
| Cancellation | ***F* (7, 98) = 2.37, *p* = .028** | | | |  | ***F* (4, 148) = 4.71 – 7.23, *p* = .000 – .001** ^a^ | | |  | **F (7, 108) = 2.45 – 3.53, *p* = .002 – .023** ^a^ | | |
| Constant |  | | -2.39 | .681 |  |  | 8.83 | .**000** |  |  | -3.59 | .535 |
| Age | .20 | | .01 | .**040** |  |  |  |  |  | .18 – .22 | .01 | .**034** |
| Gender | .08 | | .96 | .417 |  |  |  |  |  | .02 – .10 | .77 | .523 |
| SES | .05 | | .13 | .595 |  |  |  |  |  | .001 – .14 | .19 | .457 |
| T1 cancel | .17 | | .14 | .078 |  | .24 – .29 | .23 | .**001** |  | .19 – .24 | .18 | .**015** |
| Group | -.05 | | -.62 | .601 |  | -.18 – -.12 | -1.86 | .070 |  | -.09 – -.02 | -.78 | .510 |
| Regularity | .20 | | 2.77 | .**041** |  | .12 – .19 | 2.14 | .059 |  | .13 – .20 | 2.22 | .082 |
| Gp*R | .004 | | .11 | .967 |  | .03 – .15 | 2.63 | .309 |  | -.06 – .09 | .04 | .988 |
| *R*^2^ |  | |  |  |  |  |  |  |  |  |  |  |
| Numeracy-dots | | ***F* (6, 107) = 2.30, *p* = .040** | | | | | | |  |  |  |  |
| Constant |  | | -11.29 | .107 |  |  |  |  |  |  |  |  |
| Age | .31 | | .01 | .**001** |  |  |  |  |  |  |  |  |
| Gender | -.08 | | -1.18 | .397 |  |  |  |  |  |  |  |  |
| SES | .11 | | .35 | .224 |  |  |  |  |  |  |  |  |
| Group | -.14 | | -.64 | .648 |  |  |  |  |  |  |  |  |
| Regularity | .08 | | 1.23 | .415 |  |  |  |  |  |  |  |  |
| Gp*R | -.08 | | -2.64 | .382 |  |  |  |  |  |  |  |  |
| *R*^2^ | .11 | |  |  |  |  |  |  |  |  |  |  |
| **Numeracy-#s** | ***F* (6, 107) = 2.72, *p* = .017** | | | | | | | |  |  |  |  |
| Constant |  | | -10.94 | .061 |  |  |  |  |  |  |  |  |
| Age | .33 | | .01 | .**000** |  |  |  |  |  |  |  |  |
| Gender | -.18 | | -2.30 | .**048** |  |  |  |  |  |  |  |  |
| SES | .10 | | .26 | .270 |  |  |  |  |  |  |  |  |
| Group | .01 | | .17 | .884 |  |  |  |  |  |  |  |  |
| Regularity | -.06 | | -.87 | .488 |  |  |  |  |  |  |  |  |
| Gp*R | -.04 | | -1.09 | .665 |  |  |  |  |  |  |  |  |
| *R*^2^ | .13 | |  |  |  |  |  |  |  |  |  |  |
| **Flanker** |  | | | |  | ***F* (4, 148) = 2.55, *p* = .042** ^b^ | | |  | ***F* (7, 108) = 3.89, *p* = .001** ^b^ | | |
| Intercept |  | |  |  |  |  | 2.57 | .**000** |  |  | -2.64 | .092 |
| Age |  | |  |  |  |  |  |  |  | .26 | .002 | .**004** |
| Gender |  | |  |  |  |  |  |  |  | -.05 | -.16 | .596 |
| SES |  | |  |  |  |  |  |  |  | .18 | .13 | .**044** |
| T1 flanker |  | |  |  |  | .22 | .24 | .**006** |  | .32 | .34 | .**000** |
| Group |  | |  |  |  | .04 | .15 | .592 |  | -.01 | -.02 | .957 |
| Regularity |  | |  |  |  | .02 | .07 | .820 |  | -.06 | -.20 | .536 |
| Gp*R |  | |  |  |  | .11 | .83 | .171 |  | .12 | .90 | .174 |
| *R*^2^ |  | |  |  |  | .07 |  |  |  | .20 |  |  |
| **Set shifting** |  | | | |  | ***F* (4, 148) = 4.04, *p* = .004** ^b^ | | |  | *F* (7, 108) = 1.02, *p* = .422 ^b^ | | |
| Intercept |  | |  |  |  |  | 3.06 | .**000** |  |  | 3.10 | .004 |
| Age |  | |  |  |  |  |  |  |  | -.01 | .000 | .931 |
| Gender |  | |  |  |  |  |  |  |  | -.03 | -.05 | .783 |
| SES |  | |  |  |  |  |  |  |  | .11 | .04 | .262 |
| T1 set shifting |  | |  |  |  | .25 | .30 | .**001** |  | .18 | .22 | .064 |
| Group |  | |  |  |  | -.12 | -.24 | .140 |  | -.13 | -.26 | .185 |
| Regularity |  | |  |  |  | .03 | .06 | .718 |  | .03 | .06 | .789 |
| Gp*R |  | |  |  |  | -.13 | -.59 | .101 |  | -.07 | -.30 | .480 |
| *R*^2^ |  | |  |  |  | .10 |  |  |  | .06 |  |  |
| **Go / no go** | *F* (7, 27) = .59, *p* = .762 | | | |  | ***F* (4, 148) = 14.31, *p* = .000** ^b^ | | |  | ***F* (7, 108) = 5.98, *p* = .000** ^b^ | | |
| Intercept |  | | 31.29 | .338 |  |  | 29.14 | .**000** |  |  | 36.60 | .**022** |
| Age | .16 | | .01 | .437 |  |  |  |  |  | -.06 | -.01 | .517 |
| Gender | -.03 | | -.84 | .867 |  |  |  |  |  | -.03 | -1.25 | .686 |
| SES | .21 | | 1.30 | .272 |  |  |  |  |  | .07 | .52 | .407 |
| T1 go / no go | .25 | | .21 | .202 |  | .52 | .58 | .**000** |  | .50 | .54 | .**000** |
| Group | .01 | | .35 | .945 |  | .18 | 6.66 | .**012** |  | .22 | 7.92 | .**013** |
| Regularity | -.04 | | -1.18 | .830 |  | -.01 | -.34 | .907 |  | -.03 | -1.28 | .703 |
| Gp*R | .12 | | 6.67 | .537 |  | .03 | 2.40 | .683 |  | .07 | 5.37 | .424 |
| *R*^2^ | .13 | |  |  |  | .28 |  |  |  | .28 |  |  |

*Note*. ^a^ Pooled results of multiple imputation analysis. ^b^ Worst-case-scenario analysis.
